# The Five Canadas of Climate Change: Using audience segmentation to inform communication on climate policy

**DOI:** 10.1371/journal.pone.0273977

**Published:** 2022-11-23

**Authors:** Marjolaine Martel-Morin, Erick Lachapelle

**Affiliations:** Department of Political Science, Université de Montréal, Montreal, Quebec, Canada; LUMSA: Libera Universita Maria Santissima Assunta, ITALY

## Abstract

This study examines how unique audience segments within the Canadian population think and act toward climate change, and explores whether and how the level of audience engagement moderates the effect of various messages on support for climate policy. Drawing on a random probability sample of Canadian residents (N = 1207) conducted in October 2017, we first identify and describe five distinct audiences that vary in their attitudes, perceptions and behaviours with respect to climate change: the Alarmed (25%), Concerned (45%), Disengaged (5%), Doubtful (17%) and Dismissive (8%). We then explore how each segment responds to different messages about carbon pricing in Canada. We find that messages alluding to earmarking (i.e., “Invest in solutions”) or leveling the playing field for alternative energy sources (i.e., “Relative price”) increase support for a higher carbon price among the population as a whole. However, these messages decreased support for carbon pricing among more engaged audiences (e.g., Alarmed) when a low carbon price was specified to the respondent. Meanwhile, the “Relative price” is the only message that increased policy support among less engaged audiences–the Concerned and the Doubtful. In addition to highlighting the importance of tailoring and targeting messages for differently engaged segments, these results suggest that communicating around the specific consequences of carbon taxes for the prices of some goods may be a fruitful way to enhance support for carbon taxes among relatively less engaged audiences.

## Introduction

A well-established finding in social psychology suggests that people with different values, ideologies and worldviews process uncertain or conflicting evidence very differently [1, 2]. This research suggests that people tend to assimilate new information in a way that is consistent with their pre-existing attitudinal positions, a process referred to as “biased assimilation” [3]. The role of prior attitudes in the interpretation and assimilation of new information helps to explain why values, worldviews and ideology are so important in the formation of attitudes toward climate change and climate policy [4, 5]. In addition to being stronger predictors of engagement than socio-demographic characteristics and knowledge [6, 7], values, ideologies and worldviews act as perceptual filters in the process of interpreting information about climate change, thus making them a crucial component of climate communication [8]. This phenomenon of biased assimilation and motivated reasoning more generally significantly undermines any hope of generating a “one-size-fits-all” approach to climate change communication that is expected to produce change in public opinion and behaviours [9].

In response to these challenges, many scholars have emphasized the importance of knowing one’s audience and tailoring communication to fit the audiences’ preexisting dispositions and needs [9–11]. An increasingly common approach used to identify specific targets is audience segmentation, which aims to identify subgroups that share similar characteristics in terms of values, motivations, beliefs and behaviours within a population [12, 13]. While the use of audience segmentation in the context of climate change is growing [14–16], to our knowledge, there exists no detailed audience segmentation of the Canadian population in the peer-reviewed literature. Research on Canadian climate change attitudes is relatively small but growing. This literature has examined climate change perceptions across ideological [17], partisan [18], and geographic [19] dimensions, demonstrating substantial heterogeneity in Canadian public opinion. Other studies are more focused on mitigation policy preferences [20], attitudes toward energy technologies [21] and communication strategies around proposed energy infrastructure [22]. Yet little is known as to whether and how communication strategies ought to be adapted to speak to the different audiences and interpretative communities within this geographically large and culturally diverse top 5 fossil-fuel producing country. This remains an important limitation, as better integration of the principles of effective communication could potentially improve climate change communication across diverse audiences, especially when applied to controversial climate policies that are debated in public discourse.

To address these gaps, our study examines how unique audiences think and act toward climate change in Canada and explores whether and how the level of audience engagement moderates the effect of message framing on support for carbon pricing, a policy that has received substantial academic attention while generating much political debate. The aim pursued is twofold. First, to identify and describe climate change audience segments in Canada and, second, to assess whether emphasis-framing effects around carbon pricing vary as a function of audience segment. To answer these questions, we examine data from a random probability survey of adult Canadians conducted in October 2017. Building on the pioneering approach developed by the Yale/George Mason University Program on Climate Communication [15], we apply Latent Class Analysis modeling to create a novel typology of Canadian public opinion regarding climate change. We find that the Canadian population can be divided into five distinct segments, offering potential targets for climate change communication. These audience segments range along a continuum of knowledge, attitudes and behaviours, from the Alarmed (who accept climate change as a serious problem and take personal and political action to counter it) to the Dismissive (who reject the reality of climate change and oppose action). We then examine a factorial 2 X 4 survey experiment embedded in the same survey in order to explore whether audience segments moderate the effect of alternative messaging strategies for communicating carbon pricing in Canada. Overall, our results suggest that Canadians vary significantly in their level of engagement with climate change and that some messages may be better suited at speaking to specific audiences than others.

The remainder of the paper is organized as follows. We begin by examining the literature on climate change audience segments and carbon pricing communication. After outlining our research design, we present the results of our audience segmentation and survey experiment. We conclude by presenting the potential implications of our findings and proposing some new avenues for further research.

### Audience segmentation for climate change

Perhaps the best-known and long-standing effort at climate change audience segmentation to date is found in the “Global Warming’s Six Americas’’ studies realized by the Yale and George Mason Program on Climate Change Communication [15]. To identify the six audience segments, 36 variables assessing climate change motivations, behaviours and preferred societal responses of a nationally representative survey of U.S. residents were subjected to Latent Class Analysis. The six audiences identified by this method reflect quantitative shifts from high to low levels of concern, issue engagement and degree of certainty that global warming is occurring. Though some scholars have raised important concerns regarding the limits of audience segmentation as a tool of social marketing [23], the use of audience segmentation by climate change researchers has grown considerably [13]. For instance, the Global Warming’s Six Americas’ model has now been adapted and extended to India [24], Australia [14, 25, 26], Germany [16], Singapore [27], the Netherlands [28] and New Zealand [29]. Segmentation analyses have also been applied to other environmental issues, such as environmental protection [30] and worldviews [31], energy-related behaviours [32], and recycling attitudes [33] and behaviours [34]. Given the central role ideologies, values and worldviews play in shaping individuals’ response to climate change information [1, 2, 6] audience segments could act as important moderators of message framing effects. Such framing effects are thought to occur when presenting the same information in different ways (i.e., equivalency framing) or emphasizing particular aspects of an issue over others (i.e., emphasis framing) causes citizens to alter their preferences [35–38].

Despite the implicit and explicit goal of audience segmentation studies to better communicate with specific audiences with tailored messages, there are still relatively few studies that use audience segmentation to empirically assess how specific audience segments respond to different types of climate change communications and interventions. Among the exceptions, Myers et al. [39] use the Six Americas segmentation model and demonstrate that framing climate change as a health issue elicited positive emotional reactions (e.g., hope) across a range of audiences, with such effects increasing in size when comparing the most dismissive to the most concerned audience segments. As a result, they suggest that a public health frame might bolster support for climate change policy. However, this study also found that a national security frame produced an unanticipated boomerang effect on the relatively less engaged audiences (i.e., Doubtful and Dismissive segments), highlighting the propensity of different audiences to react in a non-uniform manner to a given message. In another study conducted in Australia, Hine and colleagues [14] found that messages with strong negative emotive content increased adaptation intentions in all three of their segments (i.e., Dismissive, Uncertain and Alarmed), while messages focusing on local impacts were effective at increasing engagement among dismissive audiences only. While these studies underscore the importance of considering audiences in the process of climate change communication, the varying effects of message framing across audiences generally remain underexplored, especially in the Canadian context, where no study about climate change audience segments, to our knowledge, has been published to date. To the extent that public opinion on climate change varies across cultural, political and regional environments [40, 41], patterns of climate change segmentation are likely to diverge across cultural contexts [11]. As a result, the Canadian public is unlikely to segment into the same sub-populations as the U.S., German or Australian public. Furthermore, as Metag and Schäfer point out [42], existing segmentation studies differ considerably in the constructs, measures and analytical procedures used, which makes cross-study comparisons challenging. For instance, some segmentation studies are based on Latent Class Analysis [e.g., 29, 42], while others are based on Cluster Analysis [e.g., 16, 28], or Latent Profile Analysis [e.g., 14].

With these limitations in mind, the aim of this study is not to directly assess how Canadian segments compare to those found in the United-States or other countries, but rather to provide context-relevant audience insights to inform communication for specific audiences. Specifically, we extend findings from the audience segmentation literature to the case of Canada, and explore the reactivity of different segments to messages around carbon pricing.

### Communicating about carbon pricing

Carbon pricing is one of the most commonly discussed policy instruments in both political and academic circles. According to the World Bank Carbon Pricing Dashboard, no fewer than 45 national and 34 subnational jurisdictions representing one fifth of global GHG emissions are covered by some form of a carbon price as of November 2021 [43]. Despite being supported by a broad coalition of actors including international organizations, high profile economists, as well as some of the largest environmental NGOs, however, this policy approach has proven to be politically controversial across a variety of contexts [44–47]. One of the key reasons people dislike carbon taxes is because the costs they impose are highly visible and perceived to be high [48, 49]. In fact, experimental evidence suggests that support for carbon taxes decreases with rising tax levels [50–52]. To the extent that carbon prices will need to substantially increase in order to meet the goals of the Paris Agreement [53], this public opposition to higher carbon taxes remains an important political barrier, thus pointing to the potentially heightened role of policy design and communication in a context of rising carbon prices.

In order to overcome public opposition, a burgeoning literature has pointed to the potential of revenue recycling as a means of building public support for carbon taxes and carbon pricing more generally [47, 48, 50, 54–56]. In particular, a number of scholars and policy actors have identified lump-sum transfers, or equal per capita dividends, as a promising strategy for building public support [52, 57]. However, other research suggests that earmarking revenues to invest in programs and policies that strengthen and further reduce emissions may be seen as preferable from the perspective of the public. Focus groups conducted in Denmark [58], Ireland [59], and the United Kingdom [60] all showed that respondents preferred earmarking revenues over other forms of revenue recycling to support additional emissions reductions. Consistent with this, a survey conducted on a large probabilistic sample of the Canadian population revealed a clear preference for earmarking carbon price revenues to fund investments in renewable energy (51%) over tax rebates (15%), and cuts to other taxes (7%) [18]. Thus, while the literature provides suggestive evidence regarding a potential role for policy design in building public support, we know considerably less about the most effective way of communicating information about carbon taxes, as well as how different climate change audiences might react to specific features of their design.

To be sure, there is substantial evidence to suggest that information provision on the benefits of certain carbon tax designs can enhance support [48, 54, 61]. However, other research has questioned the extent to which information deficits are part of the problem, suggesting that a better strategy may be to avoid the complex task of communicating policy details altogether [62]. In fact, rather than communicating around policy design, some researchers point to informing the public of the relative costs of alternative policy measures as a means of building support for otherwise unpopular carbon taxes that are perceived as overly costly [49]. In a similar vein, if regular citizens can’t be relied on to fully understand the intricate details of policy design, they may instead be motivated to support a carbon tax based on its specific consequences for the relative prices of essential goods (like energy).

This is in line with existing research on framing effects, which suggests that the way issues are communicated–that is, the words used, the issue dimensions that are emphasized or made salient in a communicative context–can also have a meaningful and significant impact on the public’s support for policies [e.g., 63, 64]. These kinds of efforts at communicating certain aspects of carbon tax designs or highlighting the relative costs of alternative policy measures on public support for carbon pricing can be thought of as emphasis frames. In contrast to equivalency frames, which present an issue or political choice in different but logically identical ways, emphasis frames emphasize a subset of potentially relevant features of an issue over others [35, 36], thus varying how the information is presented *and* its content [38, 65]. While some scholars have argued for a narrower definition of framing that would exclude emphasis frames [38]–notably because these effects are difficult to distinguish from other communication effects–the literature suggests that the two types of influences are important in shaping citizens’ political preferences, similarly wielding on average medium-sized effects on political attitudes and emotions across studies and contexts [66].

In light of the insights provided by the literature on audience segmentation for climate change communication, we designed a survey instrument intended to help identify specific climate change audiences that exist in the Canadian population. Informed by the literature on communicating around carbon taxes, we also embedded an experiment in the same survey to assess the reactivity of climate change audiences in Canada to messages (i.e., emphasis frames) intended to build support for carbon taxes.

## Methods

In order to examine the public’s reactivity to alternative ways of communicating around carbon taxes, we analyze data drawn from a random probability sample of 1207 adult residents of Canada. This survey was approved by the University of Montreal’s Ethics Review Board (certificate CERAS-2017-18-105-D) and consent to participate was informed. The data were collected using a random digit dialing (RDD) telephone survey with a disproportionate stratified sample of adult Canadians aged 18 years and older. An overlapping dual-frame (landline and cell phone) sample was used. Interviews were conducted between 6 and 29 October 2017 in English and French. Using the American Association of Public Opinion Research method of calculating response rates (AAPOR RR3), we obtain a combined response rate of 9%, which is typical for this method of data collection and has been shown to provide valid estimates with limited bias from unit nonresponse [67].

### Audience segmentation

To segment the data into distinct audiences, we followed the approach of Maibach and colleagues [15] and used three categories of variables as criteria for the segmentation: motivations, behaviours and preferred societal responses. However, we did not measure the full 36 variables that were used in the Global Warming’s Six Americas, as research has demonstrated that valid audience segmentation models can be obtained using a subset of 15 [15] or even 4 [68] items. The questions available in our survey allowed us to measure motivations, behaviours and preferred societal responses with 13 variables, 11 of which were very similar to those used in the Six Americas (see Table 1). As shown in Table 1, three of these items were slightly adapted to the Canadian context and two others were added because they considerably improved the predictive power of our models (i.e., attitude toward the environmental movement and importance of climate change for electoral decisions).

**Table 1:** Variables used to create the segmentation (vs. items used in Global Warming’s Six Americas).

Variables used to create the segmentation	Global Warming’s Six Americas (Maibach et al. 2011)
*Motivations* • Certainty global warming is occurring • Human causation (% agree global warming is occurring and is mostly caused by human activities) • Personal risk perception • Timing of harm to Canadians • Knowledge • Climate change discussion frequency • Attitude toward the environmental movement* • Importance of climate change for electoral decisions*	*Motivations* • Certainty global warming is occurring • Human causation (% agree) • Scientific consensus • Personal risk perception • Future generation risk perception • Risk on animals and species • Timing of harm to Americans • Ability of humans to successfully mitigate climate change • Actions of individuals can make a difference • Technological optimism • Perceived impact of own mitigation actions • Impact of own actions if widely adopted in the U.S. • Impact of own actions if widely adopted in modern industrialized countries • Rating of Global warming (1 = good to 6 = bad) • Level of worry • Thought given to global warming • Need for information (4 = low need) • Personal importance of issue • Unwilling to change opinion • Personally experienced global warming • Global warming discussion frequency • Friends share views on global warming
*Behaviours* • Contacted government officials about mitigation • Rewarded/punished companies that are/are not reducing emissions • Reduced household’s use of energy^+^	*Behaviours* • Contacted government officials about mitigation • Rewarded companies that reduced emissions • Intend to reward companies that reduce emissions • Punished companies that are not reducing emissions • Intend to punish companies that are not reducing emissions • Stage of change for lowering thermostat in winter • Stage of change for using public transportation or car pool • Stage of change for walking-biking instead of driving • Stage of change for CFL use
*Preferred societal responses* • Primary responsibility for paying the financial costs of climate change^+^ • Support for holding companies accountable^+^	*Preferred societal responses* • Priority of global warming for president and Congress • Corporations should do more-less to reduce warming • Citizens should do more-less to reduce warming • Desired US effort to reduce warming, given associated costs • Contingent int’l conditions for US mitigation action (% regardless of actions in other countries)
Total : 13 variables ^+^ Adapted * Added	Total : 36 variables

We conducted a Latent Class Analysis (LCA) using Latent Gold 5.1, and used these 13 variables to submit four, five and six segment solutions to the analysis. To guard against local maximum solutions when conducting LCA, one efficient technique is to run the estimation algorithm several times with different parameter start values [15]. To address this issue and to ensure the validity and stability of our findings, we conducted the analyses using 5 000 random sets of start values and replicated each solution ten times. All three models (4-,5-, and 6-segments) replicated exactly.

Several measures can be used to identify the correct number of classes and help choose the model that best fits the data (Table 2). Classical fit indices–including the Bayesian information criterion (BIC), sample-size adjusted Bayesian information criterion (SABIC) and the consistent Akaike information criterion (CAIC)–where lower values indicate superior fit–did not converge on a single solution, which is often the case in LCA [69]. We thus performed bootstrapped likelihood ratio tests (BLRT) which provided p-values to assess whether moving from 4- to 5- and 6-segments lead to a statistically significant improvement in model fit. P-values for both the 5- and 6-class solutions were significant at p<0.001. Finally, we calculated the Bayes factor (BF) to compare the 5- and 6-class solutions. A BF of greater than 10 provides strong support for the model with fewer classes [70], pointing in our case to the selection of the 5-class model. As is recommended when fit indices provide reasonable support for one or two candidate models [69], we also looked at how the models compare to each other in terms of face validity. From this point of view, the 5-class model clearly offered the most informative and practical results. On the one hand, the four-segment model omitted the distinction between the Doubtful and the Dismissive groups, which masks some nuance, as results from our five-segment model (described below) make evident. On the other hand, the additional group created in the six-segment model is very similar to the Concerned segment we already have in the five-segment model and thus generated confusion while not substantially contributing to a better understanding of how motivations, behaviours and preferred societal responses differ across groups.

**Table 2:** Model fit statistics.

Model	LL	BIC(LL)	SABIC	CAIC	BLRT *p* value	BF
4 classes	-15721	**32939**	32268	**33149**	-	**>15.000**
5 classes	-15570	33014	**32175**	33277	**0,000**	**>15.000**
6 classes	**-15482**	33212	32205	33529	**0,000**	-

Note: LL = log-likelihood; BIC = Bayesian information criterion; SABIC = sample-size adjusted BIC; CAIC = Consistent Akaike information criterion; BLRT = bootstrapped likelihood ratio test; BF = Bayes factor. Bolded values indicate “best” fit for each statistic.

### Survey experiment

We embedded a vignette experiment in the survey, using a 2 X 4 factorial design, in which the first factor manipulated the price level of the policy, while the second manipulated different options for revenue use and messaging. Both levels in this experiment represent emphasis framing. The first factor had two levels–informing respondents that a carbon tax would result in 2 cents per litre (low cost) and 11 cents per litre (high cost) increase in the price at the gasoline pump. These prices correspond to the equivalent price per litre of gasoline resulting from a $10 carbon tax, and a $50 carbon tax, respectively, which align with the price schedule outlined by the Pan Canadian Framework on Clean Growth and Climate Change proposed by the Trudeau government in Canada at the time the survey was conducted. The second factor involved testing a number of potential options for revenue use and messaging around such policy design, as outlined in Table 3.

**Table 3:** Summary of experimental treatments and question wording.

***Options for revenue use and messaging***		***Price***	
		2 cents/litre (low)	11 cents/litre (high)
	No message	(1) At 10$ per tonne, this policy will increase the price of fossil fuels, adding about 2 cents per litre at the pump.	(2) At 50$ per tonne, this policy will increase the price of fossil fuels, adding about 11 cents per litre at the pump.
	Invest in solutions	(3) At 10$ per tonne, this policy will increase the price of fossil fuels, adding about 2 cents per litre at the pump. ***For each dollar increase it receives from this policy*, *the government will invest 1 dollar in solutions to address climate change*: *such as in clean energy*, *transit*, *and energy efficiency*.**	(4) At 50$ per tonne, this policy will increase the price of fossil fuels, adding about 11 cents per litre at the pump. ***For each dollar increase it receives from this policy*, *the government will invest 1 dollar in solutions to address climate change*: *such as in clean energy*, *transit*, *and energy efficiency*.**
	Equal dividend	(5) At 10$ per tonne, this policy will increase the price of fossil fuels, adding about 2 cents per litre at the pump. ***The atmosphere belongs to everyone*, *and a carbon price gives a signal to everybody–business and households–to reduce their carbon pollution*. *The government plans to equally distribute all of the revenue in the form of equal per capita dividends for every citizen*.**	(6) At 50$ per tonne, this policy will increase the price of fossil fuels, adding about 11 cents per litre at the pump. ***The atmosphere belongs to everyone*, *and a carbon price gives a signal to everybody–business and households–to reduce their carbon pollution*. *The government plans to equally distribute all of the revenue in the form of equal per capita dividends for every citizen*.**
	Relative price	(7) At 10$ per tonne, this policy will increase the price of fossil fuels, adding about 2 cents per litre at the pump. ***Although a carbon price makes polluting more expensive*, *it also makes things like clean energy and electric vehicles more affordable*.**	(8) At 50$ per tonne, this policy will increase the price of fossil fuels, adding about 11 cents per litre at the pump. ***Although a carbon price makes polluting more expensive*, *it also makes things like clean energy and electric vehicles more affordable*.**

Our dependent variable is an additive index of three questions assessing respondents’ overall attitudes toward carbon taxes. The first and second items constituting the index include measures of the perceived fairness and efficacy of the experimentally manipulated carbon tax description, measured with the question: “On a scale from 0 to 10, where 0 represents very unfair and 10 very fair, how fair do you think this federal carbon price is?” and “On a scale from 0 to 10 where 0 represents very ineffective and 10 very effective, how effective or ineffective do you think this federal carbon price will be at reducing emissions?” The third item measured people’s willingness to pay the carbon tax: “All things considered, would you be very willing, fairly willing, not very willing, or not at all willing to pay higher taxes in order to reduce greenhouse gas and address climate change?” Results from exploratory factor analysis suggested that these three items were not distinct enough to be discussed separately. In fact, the three items loaded onto a single factor that accounted for 71% of the variance (S1 Table in S1 File). All three questions (i.e., perceived fairness, perceived effectiveness and willingness to pay for carbon pricing) were converted on a 0–1 scale, summed and divided by three to form an index ranging from 0 (“highly unsupportive of carbon pricing”) to 1 (“highly supportive of carbon pricing”). The index had a Cronbach’s alpha of 0.79, indicating good internal consistency.

We measured several demographics, including age, gender, education, language, region, interest in politics and party identification. We also included a measure of political ideology as a covariate for this study, measured with the question “Generally speaking, do you usually consider yourself as being at the left, the right or the centre of the political spectrum?” Including a small number of covariates in the analysis of an experiment can increase the precision of estimates by reducing noise, especially when the number of observations is small, as long as covariates are selected in advance and good theoretical reasons suggest that these covariates will influence the dependent variable significantly [71]. In line with this–and considering the abundance of research suggesting that people identifying with the ideological left are more likely to support climate policies [4, 72, 73]–political ideology was included as a covariate in our models. All measures with exact wording and descriptive statistics can be found in the S1 File.

We ran one-way ANOVAs to determine if there were any significant imbalances across groups in terms of observed variables including age, education and gender, and found no significant differences, confirming experimental balance across groups on these observed characteristics (S2 Table in S1 File).

Finally, we conducted a sensitivity analysis using G*Power [74] to estimate the effect sizes that could be detected with a power of 80% given the size of our sample and an alpha level of 0.05. Following Cohen’s guidelines [75], the analysis revealed that our design was sufficiently powered to detect small framing effects (f^2^ > 0.01) across the sample as whole, small to medium effects among the Alarmed, Concerned and Doubtful segments (f^2^ > 0.04, f^2^ > 0.02 and f^2^ > 0.06 respectively) and large effects among Disengaged and Dismissive groups (f^2^ > 0.45 and f^2^ > 0.12 respectively). A recent meta-analysis [66] reporting on 237 framing effects (N = 64,083) indicated that overall framing exerted on average medium-sized effects on citizens’ political attitudes across contexts (*d* = 0.41, which is equivalent to an f^2^ of 0.04). While our analysis is sufficiently powered to detect similar medium-sized effects among the Alarmed, Concerned and Doubtful, our sample is too small to detect such effects among the Disengaged and Dismissive. We take this into account when interpreting our results.

## Results

### The Five Canadas of Climate Change

We begin with results from the audience segmentation. Our analysis identified five unique audiences within the Canadian population, each of which understands and engages with the climate change issue in their own way: the Alarmed (25%), Concerned (45%), Disengaged (5%), Doubtful (17%) and Dismissive (8%). This distribution provides a good sense of the relative size of different climate change segments in Canada, their socio-demographic makeup, as well as their key motivations, behaviours and policy preferences (S5-S8 Tables in S1 File).

The Alarmed represent about a quarter of the Canadian population. Individuals in this group are fully convinced that climate change is happening and already harming people living in Canada, believe it requires significant changes in government policy and are already taking some personal action to reduce the threat. Of all groups, the Alarmed segment is made up of the largest proportion of respondents who are very confident in their belief that Earth is warming (87%) and that such warming is mostly the result of human activities (86%). Most of them feel very (42%) or somewhat (54%) well-informed about climate change and the majority discusses climate change very (56%) or somewhat (39%) often with family and friends.

A plurality of Canadians (about 2 in 5) falls into the Concerned category. Like the Alarmed, the Concerned believe human-caused climate change is real and support climate policy, but they are substantially less certain and engaged on the issue relative to the Alarmed. For instance, while most believe that human activity is behind rising temperature on Earth (68%) and that climate change is already harming people living in Canada (64%), fewer are very (54%) confident in their climate change beliefs. The Concerned are also distinct in their level of involvement regarding the issue. As compared to the Alarmed, very few say they feel very informed about the issue of climate change (8% vs 42%) or say they discuss the issue very often (6% vs 56%). Perhaps in part because they appear to be less informed about the issue, the Concerned differ from the Alarmed in their propensity to engage in climate-friendly actions.

Meanwhile, the Disengaged are less certain of their beliefs about climate change. Relative to all other segments, they are the most likely to indicate they are unsure as to whether or not Earth is warming (23%), or to refuse to pronounce themselves on whether they are confident that climate change is happening (28%). This propensity to indicate being “not sure” is also evident on a number of other key variables, such as the timing of impacts and personal risk perceptions. Another important distinction between the Disengaged and the two most engaged segments is that they are more than two (three) times less likely than the Concerned (Alarmed) to believe that climate change is mostly caused by human activities (26%). They are also less likely than the Concerned and Alarmed to report taking action themselves to help address climate change.

In terms of more skeptical audiences, relatively few of the Doubtful are very (12%) or somewhat (56%) confident that the average temperature on Earth is rising, and they are less likely than the national average (58%) to attribute this warming to human activities (25%). In line with their skepticism regarding the existence of anthropogenic climate change, they are also less likely than the Alarmed, Concerned, and Disengaged segments to engage in actions to address the issue.

Finally, the Dismissive audience is the most convinced that climate change is not occurring with a plurality being somewhat or very confident that the phenomenon is *not* happening (48%). Consequently, all of them reject the fact that climate change is happening and that these changes are caused by human activities. No less than half of the Dismissive believe that climate change will *never* harm people living in Canada. This group is similar to the Alarmed in their feeling of being informed about the issue. When asked how informed they believe themselves to be about climate change and global warming, about 40% of them say they feel very informed. Of all groups, they are the least likely to report any form of behavioural engagement with respect to climate change.

Examining the socio-demographic makeup of the five segments provides additional information to better understand how each audience differs from another. Compared to national averages, our results show that the Alarmed are more likely to be left-wing (28% vs. 17%) women (57% vs. 51%) with a graduate or professional degree (42% vs. 31%). The Alarmed are also more likely than average to express vote intentions for the Liberal Party of Canada (36% vs 31%) or the Green Party of Canada (11% vs. 6%), and less likely to indicate support for the Conservative Party of Canada (12% vs. 26%). Alarmed are also much more likely than the national average to report being “very interested” in politics (50% vs. 32%).

At the other end of the spectrum, we find that socio-demographic distinctions are stark when looking at the Dismissive group. Compared to national averages, the Dismissive are more likely to live in Alberta (24% vs. 11%) and less likely to live in Ontario (30% vs. 38%) or Quebec (12% vs. 24%). Relative to the full sample, the Dismissive are more likely to be English-speaking (76% vs. 63%) men (84% vs. 49%), with a plurality on the right side of the political spectrum (39% vs. 11%). Similar to the Alarmed (51%), a majority of the Dismissive (57%) say they are very interested in politics. However, unlike the other audiences, they are relatively uniform in their electoral choice, with a strong majority expressing a preference for the Conservative Party of Canada (77%).

Comparing across segments, we find fewer socio-demographic features that distinguish the Concerned from the Doubtful. Not surprisingly, these two groups–which are more moderate in their positions–are more likely to be found at the center of the political spectrum (42% in both cases). A plurality of the remaining Concerned are either at the center-left (13%) or at the left (17%), while the remaining Doubtful self-identify at the center-right (13%) or right (17%). Compared to the Alarmed (42%), Concerned (32%) and Dismissive (31%), the Doubtful are less likely to have a graduate or professional degree (19%). We also found some differences in terms of the regional distribution of these audiences. Relative to the Concerned, the Doubtful are more likely to live in Alberta (19% vs. 8%), and less likely to live in Ontario (33% vs. 41%) or Quebec (21% vs. 27%).

### Communicating around carbon taxes

Next, we review results from the analysis of messaging effects with respect to carbon tax support. Before proceeding to our core empirical analysis using Ordinary Least Squares (OLS) regression modeling, we present descriptive statistics with mean value comparisons of attitudes toward the policy for each of the eight treatment groups (Table 4). Because of the highly heterogeneous distribution of the audiences–with the Concerned accounting for as much as 45% of the respondents, and the Disengaged and Dismissive making up for only 5% and 8% of the sample–the number of observations per treatment is relatively low for some segments, especially the Disengaged and Dismissive (with 0 to 16 respondents per treatment). This is consistent with the results of our post hoc sensitivity analysis–which indicated that our design was sufficiently powered to detect small to medium effects among the Alarmed, Concerned and Doubtful segments, but not among the Disengaged and Dismissive. Given this, we focus on the Alarmed, Concerned and Doubtful when looking at between-segment differences in message responses.

**Table 4:** Mean level of attitudes toward carbon taxes, by audience segment and experimental treatment (mean/standard deviation, N in parentheses).

		National average	Alarmed	Concerned	Disengaged	Doubtful	Dismissive
**2 cents/litre (low)**	No message	.48/.25	.64/.19	.52/.19	.40/.35	.30/.21	.16/.22
		(131)	(34)	(58)	(4)	(21)	(14)
	Equal dividend	.46/.27	.65/.24	.51/.20	.49/.33	.33/.22	.05/.07
		(133)	(32)	(57)	(6)	(26)	(12)
	Invest in solutions	.44/.28	.56/.27	.52/.22	.27/.18	.32/.26	.09/.11
		(137)	(37)	(53)	(5)	(31)	(11)
	Relative price	.48/.25	.59/.25	.52/.22	.60/.13	.37/.23	.12/.2
		(131)	(36)	(58)	(3)	(26)	(8)
**11 cents/litre (high)**	No message	.42/.27	.56/.27	.44/.26	.	.32/.22	.15/.2
		(141)	(29)	(72)	(0)	(28)	(12)
	Equal dividend	.46/.27	.57/.22	.53/.23	.33 /.30	.39/.27	.21/.25
		(137)	(22)	(68)	(7)	(24)	(16)
	Invest in solutions	.48/.27	.55/.22	.58/.23	.57/.08	.23/.16	.09/.11
		(138)	(36)	(69)	(2)	(20)	(11)
	Relative price	.53/.24	.66/.19	.54/.19	.39/.39	.45/.21	.09/.23
		(136)	(36)	(79)	(2)	(19)	(9)

Causal effects between experimental conditions were assessed using OLS regressions on our dependent variable. To make interpretation more straightforward, we also estimated the marginal effects of each treatment on attitudes toward carbon taxes. We began by examining how the level of policy stringency affected attitudes toward carbon taxes and whether the Five Canadas of Climate Change moderate this effect. First, we considered the effect of an 11-cent price increase on attitudes toward carbon taxes (as compared to 2 cents). As shown in the S1 File (Model 1 of S3 Table in S1 File), the effect of policy stringency was very small (0.001) and non-significant. Next, we included the Five Canadas of Climate Change in our model and examined the extent to which audience segments helped predict overall attitudes. As model 2 of S3 Table in S1 File demonstrates, the Five Canadas were significant predictors of attitudes toward the policy, with positive coefficients indicating that the model predicted more supportive attitudes in each given group as compared to the reference category (i.e., the Dismissive). Belonging to the Doubtful increased the level of supportive attitudes by about 18% compared to the Dismissive segment, while belonging to the Alarmed increased the level of supportive attitudes by 42% relative to the Dismissive. In comparison, moving from the far right to the far left on the ideological scale had a 14-percentage point effect on our dependent variable. The effect of the Five Canadas of Climate Change on attitudes toward carbon taxes is thus three times greater than that of political ideology. Adding the Five Canadas to the base model significantly improved model fit, with an adjusted r-squared increasing from 0.12 in the base model to 0.30 in the fully specified model, *F* (4, 922) = 64.10, *p* < 0.001.

To test the potential of policy stringency to affect audiences in different ways, we added interaction terms, multiplying segments by price levels (Model 3 of S3 Table in S1 File). Interaction terms between segments and price levels were very close to zero and failed to reach statistical significance, suggesting that moving stringency from a low (2 cents) to a relatively higher (11 cents) increase in the gasoline price at the pump left attitudes unchanged across segments.

Next, we examined whether emphasizing different options for revenue use and messaging affected attitudes toward the policy and whether the Five Canadas of Climate Change responded differently to these treatments, both at low and higher levels of policy stringency (Table 5). Our results suggest that *overall* messaging effects only occurred when policy stringency was relatively higher. As Model 1 (Table 5) shows, none of the experimentally manipulated messages reached statistical significance when policy stringency was low. However, when the policy was said to increase the gasoline price by a higher amount (i.e., 11 cents), both the “Invest in solutions” and the “Relative price” messages significantly affected the dependent variable, respectively increasing the level of supportive attitudes by 9% and 11%. The “Equal dividend” message had no effect on attitudes at either level of policy stringency.

**Table 5:** Effect of emphasis framing on attitudes toward carbon taxes, conditional on the Five Canadas.

	Model 1			Model 2		Model 3	
	2 cents		11 cents	2 cents	11 cents	2 cents	11 cents
Invest in solutions	-.054		.088**	-.044	.07*	-.09	-.092
	(.032)		(.032)	(.027)	(.03)	(.088)	(.101)
Equal dividend	-.015		.058	-.011	.057	-.129	.023
	(.032)		(.033)	(.028)	(.03)	(.088)	(.095)
Relative price	-.021		.106**	-.024	.095**	-.034	-.1
	(.032)		(.033)	(.028)	(.03)	(.098)	(.107)
Doubtful				.195***	.169***	.095	.089
				(.041)	(.045)	(.077)	(.086)
Disengaged				.372***	.251*	.286*	.152
				(.075)	(.11)	(.134)	(.129)
Concerned				.375***	.337***	.315***	.21**
				(.038)	(.041)	(.067)	(.078)
Alarmed				.442***	.401***	.428***	.353***
				(.04)	(.045)	(.07)	(.086)
Invest*Doubtful						.093	.031
						(.109)	(.122)
Invest*Concerned						.098	.271*
						(.098)	(.109)
Invest*Alarmed						-.017	.072
						(.102)	(.118)
Equal*Doubtful						.175	.063
						(.11)	(.118)
Equal*Concerned						.105	.062
						(.097)	(.103)
Equal*Alarmed						.125	-.05
						(.103)	(.117)
Relative*Doubtful						.112	.262*
						(.119)	(.129)
Relative*Concerned						.022	.201
						(.107)	(.114)
Relative*Alarmed						-.07	.184
						(.111)	(.122)
Ideology	.083***		.066***	.045***	.028**	.048***	.031***
	(.009)		(.009)	(.009)	(.009)	(.009)	(.009)
Constant	.325***		.278***	.075*	.074	.115	.16*
	(.029)		(.031)	(.038)	(.041)	(.06)	(.072)
Obs.	458		471	458	471	458	471
Adjusted R-squared	0.141		0.111	0.348	0.265	0.374	0.292

*** p < .001

** p < .01

* p < .05

Note: values are unstandardized regression coefficients with standard errors in parentheses. Constant represents intercepts of price level at 2 cents and Dismissive segment when segments are included. Attitudes toward carbon taxes range from 0 (highly unsupportive) to 1(highly supportive). Ideology is coded 0 (right), 1 (center right), 2 (center), 3 (center left) and 4 (left).

**Fig 1:**
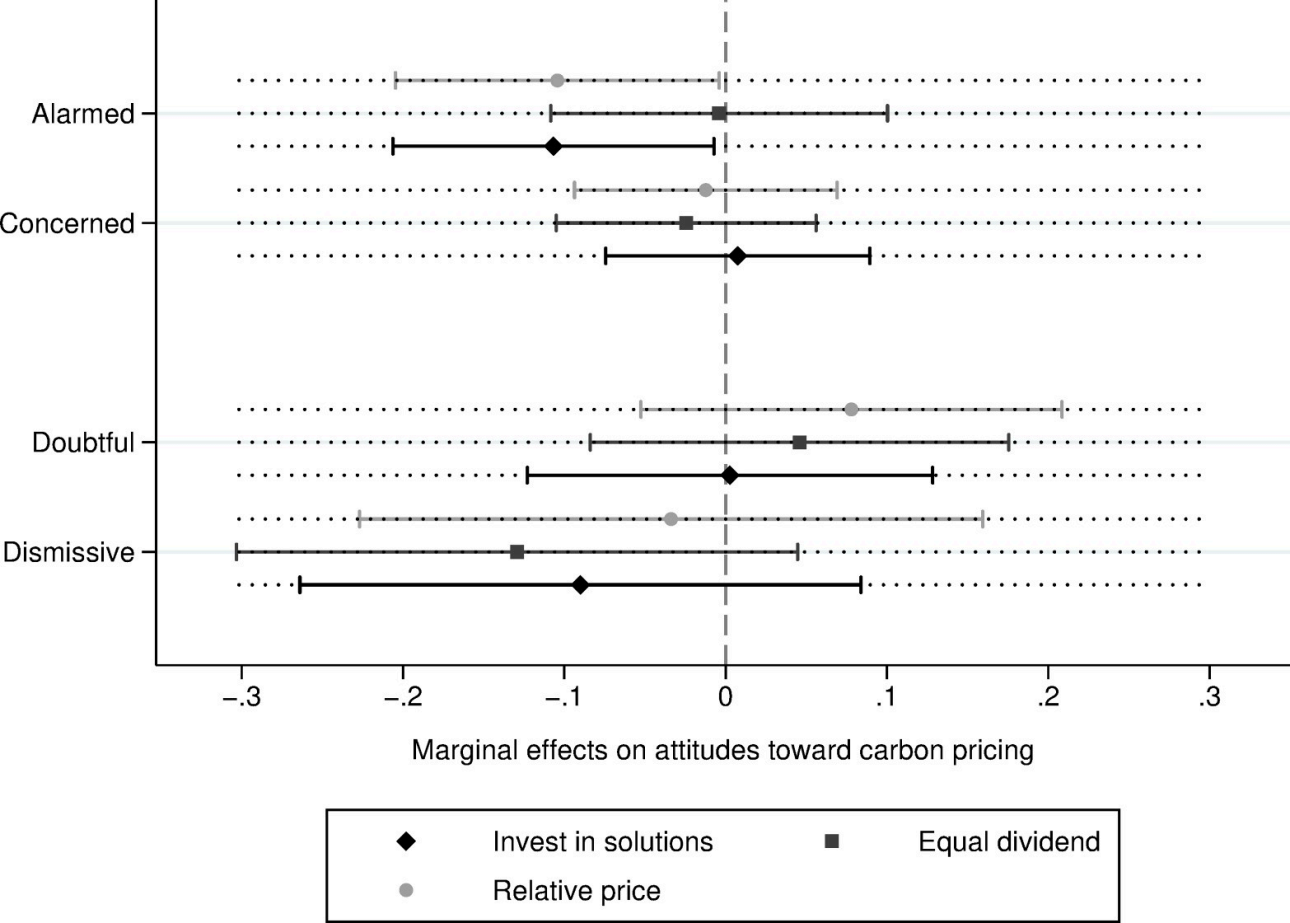
Marginal effect of emphasis framing on attitudes toward carbon pricing under low price specification, conditional on the Five Canadas (95% Cis).

To examine how each segment responded to these messages, we again included interaction terms, this time multiplying the second factor (i.e., options for revenue use and messaging) by the five audiences. While results from Table 5 suggested that none of the messages had a main effect on attitudes when stringency was low (2 cents), Fig 1 shows that the “Invest in solutions” and the “Relative price” messages had an influence on attitudes at a lower level of policy stringency, but only among the Alarmed. Rather than increasing support for the policy, however, these messages *decreased* support by about 11 percentage points among this relatively more engaged segment.

**Fig 2:**
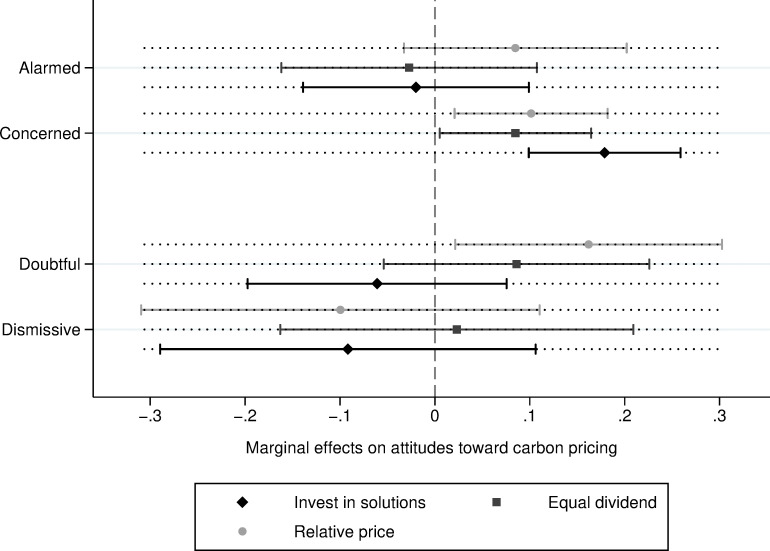
Marginal effect of emphasis framing on attitudes toward carbon pricing under high price specification, conditional on the Five Canadas (95% Cis).

Turning to the analysis of messaging effects under the higher price (11 cents per litre) condition, the “Invest in solutions” message increased carbon tax support among the Concerned, though it had no effect on the other segments (Fig 2). Despite this, the green reinvestment message was very impactful among the Concerned: the Concerned exposed to the “Invest in solutions” message scored 18% higher on the attitude index relative to the Concerned in the “no message” group. The “Equal dividend” and the “Relative price” messages were also effective with the Concerned under the moderate price treatment, increasing the level of supportive attitudes by about 9% and 10% respectively. Finally, exposure to the “Relative price” message also led to a 16% increase in positive attitudes toward carbon taxes among Doubtful.

## Discussion

Our analysis of the data measuring how Canadians think and act toward climate change revealed that the Canadian population can be divided into five distinct segments: the Alarmed (25%), Concerned (45%), Disengaged (5%), Doubtful (17%) and Dismissive (8%). These results are broadly consistent with Global Warming’s Six Americas [15] and akin to typologies identified in Australia [12, 14] and Germany [16], though we did not find a Cautious segment in Canada. The Cautious–who believe in climate change but view it as a distant problem–comprised between a quarter and a fifth of the American, Australian and German populations at the time these studies were conducted. The most recent segmentation in Australia [14], however, found three groups rather than six: the Alarmed, the Uncommitted and the Dismissive. While a Dismissive group was found in the U.S, Australia and Canada (as our data showed), this group was notably absent in the segmentation analysis conducted in Germany, reflecting differences in climate change audiences across countries.

To be sure, the potential for cross-national comparisons remains limited, as different variables were used to create the segmentations and some studies draw on data collected several years apart (e.g., the German segmentation draws on data collected in 2011, whereas our data were collected in 2017). Although caution should be exercised in comparing segments across studies, the fact that we did not find a Cautious segment similar to that identified in the United States is broadly consistent with previously reported differences in attitudes toward climate change across Canada and the United States [e.g., 18]. With respect to the Cautious, Maibach and colleagues [15] describe this segment as individuals who believe in climate change but who tend see it as a distant problem, hence not feeling the urgency to take action. In the spring of 2015, the Pew Research Center reported that, while Canadians and Americans expressed relatively high levels of concern about climate change (64% vs. 59%), the latter were considerably less likely than the former to believe that climate change is harming people around the world now (41% vs. 56%) [76]. To some extent, the absence of a Cautious segment in Canada reflects these differences in risk perceptions.

These differences notwithstanding, our results are similar to existing studies using climate change audience segmentation techniques, which demonstrate the predictive power of audience segments relative to demographics and other political variables found to be important correlates of climate policy support in the literature [4]. Explaining 26% of the variance in attitudes toward carbon pricing, the predictive power of our segmentation is consistent with previous studies [12, 15] where audience membership explained between 14% and 55% of the variance in responses to climate change.

In terms of the overall impact of messages on climate policy attitudes, we found that both the “Invest in solutions” and “Relative price” messages had, across the sample as a whole, a positive impact on carbon pricing attitudes when policy stringency was higher. This result echoes previous experimental evidence of framing effects found in other studies. For instance, Beiser-McGrath and Bernauer [50] employed a conjoint design and found that information provision about revenue use significantly increased public support for a carbon tax in the United States and Germany, but similar to our study, these framing effects were most pronounced at relatively higher levels of policy stringency. This finding may suggest that communication of carbon tax benefits is especially important as carbon prices become more substantial, or as concerns about the cost of living rise. Moreover, it is worth noting that of the three options for revenue use and messaging tested, only the “Equal dividend” message had no *overall* effect on attitudes. This is at odds with research highlighting the role of equal per capita dividends in building support for carbon taxes [52, 57] though it is in line with evidence suggesting that the public generally likes the idea of earmarking revenues to support additional emissions reductions [18, 58–60]. It is also possible that this frame was less effective because of the additional information that was introduced to accompany the treatment (i.e., “The atmosphere belongs to everyone, and a carbon price gives a signal to everyone—business and households—to reduce their carbon pollution”). While we included this information in an attempt to make the treatment stronger, we acknowledge it could also have weakened the treatment if respondents reacted negatively to the additional text.

Beyond these aggregate effects, we also found that emphasis framing affected the Five Canadas in very different–and sometimes unintended–ways. The “Invest in solutions” and the “Relative price” messages had a *negative* impact on attitudes toward carbon taxes among the Alarmed when policy stringency was low, and a positive impact on attitudes among the Concerned when policy stringency was higher. While all messages were effective with the Concerned, the “Relative price” was the only message positively affecting both the Concerned and the Doubtful. These findings echo recent research looking at the role of political variables in moderating the effect of carbon tax design on levels of policy support, which found heterogenous sub-group effects across individuals with different political ideologies [52] and partisan identities [56, 77]. However, these studies focused specifically on revenue recycling options (e.g., tax rebates, deficit reduction, support for renewable energy technology) and used unidimensional segmentation criteria.

In contrast, we examined information provision and relative price effects using broader segmentation criteria, and showed that communicating around the specific consequences of carbon taxes for the price of essential goods (like energy) may be more effective than communicating around policy design, especially among less engaged audiences (i.e., the Concerned and Doubtful segments). That these framing effects were mostly found among moderately engaged groups echoes Zaller’s [78] two-moderator model of persuasion, and is in line with research by Chong and Druckman [79] highlighting the role of strongly held prior attitudes in moderating framing effects. Both of these studies suggest that framing effects are most likely to be found among audiences with relatively weaker opinions.

A negative effect of the “Invest in solutions” and “Relative price” messages among the Alarmed is also broadly consistent with previous studies documenting “boomerang effects”, occurring when exposure to a given message moves beliefs in a direction opposed to the original intent [39, 80]. However, in contrast to these studies–which typically found boomerang effects among groups that were more skeptical of climate change—our results demonstrate that boomerang effects may also occur among the most Alarmed segments of the population.

This result among the Alarmed could be explained by the relative sophistication of this audience. Recall that of all segments, the Alarmed are most engaged with climate change and are much more likely than national averages to report being both knowledgeable about the climate change issue, and very interested in politics. As a result, the Alarmed may be more motivated by accuracy goals and to scrutinize the credibility of messages, thus making them less inclined to believe that such a low (2 cents per litre) carbon price can have any real impact on green infrastructure or the relative price of different energy technologies [c.f., 3]. To test this explanation, we created a dummy variable equal to 1 for respondents who received the "Invest in solutions" or the "Relative price" treatment (i.e., those with a negatively signed coefficient at a low price stringency) and 0 for those who received the "Equal dividend" treatment. We then examined whether these treatments had a different effect on perceptions of policy effectiveness, and whether policy stringency moderated this effect. As shown in the S4 Table in S1 File, we found a negative interaction effect between the "Invest in solutions" and the "Relative price" messages and the level of policy stringency. Consistent with the idea that the Alarmed are more sophisticated and therefore more likely to scrutinize the coherence of carbon pricing messages, we found that messages alluding to substantial earmarking or leveling the playing field for alternative energy sources decreased perceptions of policy effectiveness among the Alarmed, but only under the lower price treatment.

Overall, our results suggest that attempts at devising uniform, one-size-fits-all communication strategies around climate policy are likely to face important challenges. For instance, our findings suggest that governments and communicators should be careful when communicating with more sophisticated and engaged climate change audiences (like the Alarmed), who may be more motivated by accuracy goals when processing messages around climate change. As a result, careful consideration ought to be given to the credibility of messages, so as not to alienate more engaged audiences as they seek to win the support of less engaged groups. More broadly, the different framing effects found at different levels of policy stringency further suggest that the overall cost structure is important when crafting messages about climate policy. As the costs of climate policy and concerns with cost of living evolve over time, communications may need to be adapted so they better fit the context in which messages are received, while meeting the informational needs and motivations of different audiences.

## Conclusions

Analyzing data from a random probability survey of adult Canadians collected in October 2017, this study examined how unique audience segments within the Canadian population think and act toward climate change and explored whether and how the level of audience engagement moderates the effect of various messages on support for climate policy. We found that the Canadian population can be divided into five distinct segments, offering potential targets for climate change communication: the Alarmed (25%), Concerned (45%), Disengaged (5%), Doubtful (17%) and Dismissive (8%). These audiences reacted to emphasis framing in very different—and sometimes unintended—ways. In particular, “Invest in solutions” and “Relative price” messages had a negative impact on attitudes toward carbon taxes among more engaged audiences (i.e., the Alarmed) when policy stringency was low, and a positive impact on attitudes among the Concerned when policy stringency was higher. The “Relative price” was the only message positively affecting both the Concerned and the Doubtful, suggesting that communicating around the specific consequences of carbon taxes for the prices of essential goods (like energy) may be a fruitful way to broaden support for carbon pricing among moderately engaged audiences. This may particularly be important as the costs of climate policy rise as well as in contexts where cost of living considerations are top of mind.

In extending findings from the audience segmentation literature to the Canadian case, and by exploring how each segment responds to different messages about carbon taxes, our study helps better understand heterogeneity in Canadian attitudes toward climate change as well as the potential promise and pitfalls involved when attempting to communicate about a controversial policy across a broad range of differently engaged audience segments. In this sense, our study contributes to the literature on climate change policy and communication by highlighting the importance of audience-based data in supporting climate research, policy and communication and offering a starting point for further work aimed at developing messages that speak to different climate change audiences.

While exploratory in nature, our analysis is limited in a number of important respects. First, our findings are based on a relatively small sample of the five audience groups. In analyzing how these audiences respond to the treatments tested in our experiment, we had to rely on a small number of observations. Caution should be exercised when interpreting these results, notably for segments for which we have the smallest samples (i.e., Disengaged and Dismissive audiences). The null results reported in this analysis might reflect a lack of statistical power, particularly for these smaller audiences. In order to detect medium-sized effects with a power of 80% and an alpha level of 0.05, it would require a sample of 179 respondents in each audience segment. Given that this is the first segmentation study of its kind for Canada, it was difficult to anticipate the size of the audiences a priori. While we were able to meet this threshold for three segments (i.e., the Alarmed, Concerned and Doubtful), more research with larger samples would be required to determine the effectiveness of different frames across all audiences. For instance, future research could use data available from social media platforms as an exciting (and less costly) research possibility to classify climate change audiences from larger samples.

Second, our analysis provided a high-resolution portrait of attitudes toward climate change at one specific point in time, offering little insight into the evolution of climate opinions and audiences, as well as limited information on the durability of the observed framing effects. Many things have changed since 2017 in terms of national and international discourses on climate change, likely bringing about changes in the composition of the segments. For instance, the Six America’s studies in the United States documented a clear trend toward rising Alarm in the United States [81]. Although we can only speculate as to whether Canada followed a similar trend, our study provides a baseline against which future research can be compared. Other studies could replicate this segmentation using longitudinal data to provide a more dynamic picture of Canadian attitudes towards climate change. Finally, there are other important messages, and other factors that may condition messaging effects that were not included in this analysis, such as the source of the message and the availability of competing frames or arguments, which we leave for future work.

Building on this work, future analyses might go further and examine patterns of information behaviours and media use across audiences. Our study also calls for more research on climate change audiences using different types of research designs, including longitudinal analyses. Future research might also examine whether and how the source of the message influences messaging effects among different audiences and explore how the latter respond to frames as they compete with each other. These kinds of extensions will help to further our collective understanding of how messages interact with different audience characteristics, providing more insight into what to consider when attempting to communicate about controversial policies with a heterogeneous public.

## Supporting information

S1 File (DOCX)
