# Supplementary material for: The Five Canadas of Climate Change: Using audience segmentation to inform communication on climate policy
Source: PLoS One. 2022 Nov 23;17(11):e0273977. doi: 10.1371/journal.pone.0273977 (PMC9683580; doi:10.1371/journal.pone.0273977)
Supplement: S1 File — (DOCX) [file pone.0273977.s001.docx]

**S1 File**

The Five Canadas of Climate Change: Using Audience Segmentation to Inform Communication on Climate Policy

PLOS One

**Measures and experimental balance across treatments**

**Table S1. Factor loadings for overall attitudes toward carbon taxes**

| Measures of overall attitudes  toward carbon taxes (∝=.79) | Factor loadings  (one-factor solution) |
| --- | --- |
|  |  |
| Willingness to pay the carbon tax | .56 |
|  |  |
| Perceived effectiveness | .57 |
|  |  |
| Perceived fairness | .60 |
|  |  |
|  |  |

**Table S2.** **Analyses of variance: demographic characteristics by experimental treatment (mean/standard deviation, N in parentheses)**

|  |  | No message | Equal dividend | Invest in solutions | Relative  price | P-values |
| --- | --- | --- | --- | --- | --- | --- |
| **2 cents/litre (low)** | Gender | .51/.50 (146) | .54/.50 (149) | .55/.50  (148) | .47/.50 (146) | .4515 |
|  | Education | 2.67/1.23  (150) | 2.64/1.27 (151) | 2.60/1.27 (151) | 2.56/1.32 (151) | .8862 |
|  | Age | 54/18 (150) | 55/17 (151) | 56/17 (151) | 52/18 (151) | .3113 |
| **11 cents/litre (high)** | Gender | .50/.50 (149) | .49/.50 (142) | .48/.50  (147) | .53/.50  (147) | .8158 |
|  | Education | 2.75/1.20  (151) | 2.66/1.28 (153) | 2.47/1.24 (150) | 2.75/1.24 (150) | .1717 |
|  | Age | 54/17 (151) | 54/19 (153) | 54/19 (150) | 53/18 (150) | .9432 |

**Regression tables**

**Table S3. Price effect on attitudes toward carbon taxes, by audience segment**

|  | Model 1 | Model 2 | Model 3 |
| --- | --- | --- | --- |
|  |  |  |  |
| High price | .001 | .004 | .055 |
|  | (.016) | (.015) | (.049) |
| Doubtful |  | .178*** | .197*** |
|  |  | (.03) | (.043) |
| Disengaged |  | .33*** | .383*** |
|  |  | (.062) | (.078) |
| Concerned |  | .355*** | .386*** |
|  |  | (.028) | (.039) |
| Alarmed |  | .424*** | .453*** |
|  |  | (.03) | (.041) |
| High price*Doubtful |  |  | -.037 |
|  |  |  | (.06) |
| High price*Disengaged |  |  | -.133 |
|  |  |  | (.13) |
| High price*Concerned |  |  | -.059 |
|  |  |  | (.054) |
| High price*Alarmed |  |  | -.06 |
|  |  |  | (.057) |
| Ideology | .073*** | .035*** | .036*** |
|  | (.007) | (.006) | (.006) |
| Constant | .322*** | .092*** | .066 |
|  | (.018) | (.026) | (.036) |
| Obs. | 929 | 929 | 929 |
| Adjusted R-squared | 0.116 | 0.298 | 0.303 |
|  | | | |
| **** p<.001, ** p<.01, * p<.05*  Note: values are unstandardized regression coefficients with standard errors in parentheses. Constant represents intercepts of price level at 2 cents and Dismissive segment when segments are included. Attitudes toward carbon taxes range from 0 (highly unsupportive) to 1(highly supportive). Ideology is coded 0 (right), 1 (center right), 2 (center), 3 (center left) and 4 (left). | | | |

**Table S4. Effect of emphasis framing on perceptions of effectiveness among the Alarmed**

|  | | Coef. | St.Err. | t-value | | | p-value | [95% Conf | | Interval] | Sig |  |
| --- | --- | --- | --- | --- | --- | --- | --- | --- | --- | --- | --- | --- |
| Invest solutions/Relative price vs. Equal dividend | | .064 | .060 | 1.06 | | .290 | | -.055 | | .184 |  |  |
| Low price | | .114 | .069 | 1.64 | | .102 | | -.023 | | .251 |  |  |
| Invest solutions/Relative price * low price | | -.162 | .081 | -2.01 | | .046 | | -.322 | | -.003 | * |  |
| Ideology | | .059 | .015 | 4.02 | | .000 | | .030 | | .089 | *** |  |
| Constant | | .310 | .065 | 4.77 | | .000 | | .182 | | .437 | *** |  |
|  | | | | | | | | | | | | |
| Mean dependent var | .512 | | | | SD dependent var | | | | .255 | |  |  |
| R-squared | .097 | | | | Number of obs | | | | 191.000 | |  |  |
| F-test | .992 | | | | Prob > F | | | | .001 | |  |  |
| Akaike crit. (AIC) | .980 | | | | Bayesian crit. (BIC) | | | | 26.241 | |  |  |
| **** p<.001, ** p<.01, * p<.05* | | | | | | | | | | |  |  |

**Audience segments**

Our analysis of the way in which Canadians think and act towards climate change revealed that the Canadian population can be divided into five distinct segments: the Alarmed (25%), Concerned (45%), Disengaged (5%), Doubtful (17%) and Dismissive (8%).

The Alarmed are very confident in their belief that the planet is warming (87%) and that such warming is mostly the result of human activity (86%). Of all groups, they are the most likely to say they have already taken some personal action to reduce the threat of climate change.

The Concerned believe that human activity is responsible for rising global temperatures (68%), but they are less likely than the Alarmed to be very (54%) confident in this belief. Relative to the Alarmed, the Concerned are also less likely to engage in climate-friendly action.

The Disengaged are less certain of their beliefs about climate change. About one in four are unsure as to whether or not the planet is warming (23%), or refuse to pronounce themselves on whether they are confident that climate change is happening (28%). The Disengaged are less likely than the Concerned and Alarmed to report taking action themselves to help address climate change.

Relatively few of the Doubtful are very (12%) or somewhat (56%) confident that the average temperature on the planet is rising, and only about 1 in 4 (25%) attribute this warming to human activity. Compared with the Alarmed, Concerned, and Disengaged segments, the Doubtful are less likely to engage in actions to address the issue.

Finally, the Dismissive audience is the most convinced that climate change is not occurring, with a plurality being somewhat or very confident that the phenomenon is not happening (48%). Of all groups, they are the least likely to report any form of behavioural engagement with climate change.

The following tables provide additional information about the motivations (Table S5), behaviours (Table S6), preferred societal responses (Table S7) and demographic characteristics (Table S8) of the audience segments. All data in the Tables are percentages.

**Table S5. Motivations, by audience segment**

|  | Alarmed | Concerned | Disengaged | Doubtful | Dismissive | Avg. |
| --- | --- | --- | --- | --- | --- | --- |
| **How confident are you that the average temperature on Earth is/is not increasing?** |  |  |  |  |  |  |
| Very confident it is increasing | 87 | 54 | 28 | 12 | 13 | 51 |
| Fairly confident it is increasing | 12 | 42 | 19 | 56 | 12 | 33 |
| Not too confident it is increasing | 1 | 2 | 8 | 11 | 6 | 4 |
| Not confident at all it is increasing | 0 | 0 | 7 | 0 | 4 | 1 |
| Not sure/refused | 0 | 2 | 28 | 8 | 4 | 4 |
| Not confident at all it is not increasing | 0 | 0 | 6 | 0 | 4 | 1 |
| Not too confident it is not increasing | 0 | 0 | 1 | 4 | 9 | 1 |
| Fairly confident it is not increasing | 0 | 0 | 0 | 9 | 22 | 3 |
| Very confident it is not increasing | 0 | 0 | 3 | 1 | 26 | 2 |
| **Is the earth getting warmer mostly because of human activity such as burning fossil fuels, or mostly because of natural patterns in the earth's environment?** |  |  |  |  |  |  |
| Mostly human activity | 86 | 68 | 26 | 25 | 0 | 58 |
| Mostly natural patterns/a combination/not sure/refused | 14 | 32 | 74 | 75 | 100 | 42 |
| **When do you think climate change will start to harm people living in Canada?** |  |  |  |  |  |  |
| Climate change is already harming people in Canada | 84 | 64 | 29 | 22 | 3 | 55 |
| In 10 years | 8 | 16 | 24 | 12 | 1 | 13 |
| 25 years | 2 | 12 | 5 | 16 | 6 | 9 |
| 50 years | 4 | 7 | 0 | 25 | 5 | 9 |
| 100 years | 0 | 1 | 0 | 17 | 20 | 5 |
| Never | 1 | 0 | 5 | 4 | 55 | 5 |
| Not sure/refused | 2 | 1 | 38 | 4 | 10 | 4 |
| **How well informed do you believe yourself to be on the issue of climate change?** |  |  |  |  |  |  |
| Very well informed | 42 | 8 | 17 | 9 | 40 | 20 |
| Somewhat informed | 54 | 69 | 30 | 44 | 52 | 58 |
| Not too informed | 3 | 20 | 25 | 43 | 3 | 18 |
| Not informed at all | 0 | 3 | 25 | 3 | 4 | 3 |
| Not sure/refused | 0 | 0 | 3 | 1 | 1 | 0 |
| **How much do you think climate change will harm you personally?** |  |  |  |  |  |  |
| A great deal | 37 | 13 | 23 | 2 | 1 | 17 |
| A moderate amount | 42 | 43 | 24 | 21 | 2 | 35 |
| Only a little | 18 | 36 | 20 | 42 | 10 | 30 |
| Not at all | 3 | 6 | 18 | 35 | 84 | 17 |
| Not sure/refused | 0 | 2 | 16 | 0 | 3 | 2 |
| **Table S6. Behaviours, by audience segment** | | | | | | |
|  | Alarmed | Concerned | Disengaged | Doubtful | Dismissive | Avg. |
| **How often do you discuss climate change with your family and friends?** |  |  |  |  |  |  |
| Very often | 56 | 6 | 11 | 7 | 29 | 21 |
| Occasionally | 39 | 66 | 10 | 30 | 28 | 47 |
| Rarely | 5 | 25 | 24 | 41 | 28 | 23 |
| Never | 0 | 3 | 50 | 22 | 15 | 9 |
| Not sure/refused | 0 | 0 | 5 | 0 | 0 | 0 |
| **In the past year, have you, yourself… Written a letter, emailed or phoned a government official to urge them to take action on climate change?** |  |  |  |  |  |  |
| Yes | 24 | 3 | 5 | 0 | 5 | 8 |
| No | 76 | 97 | 88 | 100 | 95 | 91 |
| Not sure/refused | 0 | 0 | 8 | 0 | 0 | 0 |
| **In the past year, have you, yourself… Deliberately bought or boycotted certain products based on their environmental impact?** |  |  |  |  |  |  |
| Yes | 84 | 54 | 13 | 30 | 20 | 53 |
| No | 14 | 44 | 74 | 70 | 80 | 45 |
| Not sure/refused | 2 | 2 | 13 | 0 | 0 | 2 |
| **How important a factor is climate change and or climate policy in deciding which party gets your vote?** |  |  |  |  |  |  |
| A deciding factor | 34 | 7 | 9 | 5 | 7 | 14 |
| Somewhat a deciding factor | 51 | 62 | 10 | 20 | 13 | 46 |
| Neutral | 6 | 18 | 10 | 26 | 4 | 15 |
| Somewhat not a deciding factor | 2 | 9 | 9 | 27 | 15 | 11 |
| Not a deciding factor at all | 5 | 3 | 12 | 20 | 60 | 11 |
| Not sure/refused | 2 | 2 | 51 | 1 | 1 | 4 |
| **Thinking specifically about the environmental movement, do you think of yourself as…** |  |  |  |  |  |  |
| An active participant in the environmental movement | 36 | 13 | 15 | 10 | 6 | 18 |
| Sympathetic towards the movement, but not active | 54 | 68 | 18 | 31 | 13 | 51 |
| Neutral | 8 | 18 | 41 | 46 | 29 | 22 |
| Unsympathetic towards the environmental movement | 2 | 1 | 7 | 12 | 52 | 7 |
| Not sure/refused | 0 | 0 | 18 | 2 | 0 | 1 |
|  |  |  |  |  |  |  |
| **Table S7. Preferred societal responses, by audience segment** | | | | | | |
|  | Alarmed | Concerned | Disengaged | Doubtful | Dismissive | Avg. |
| **Who or which group should be primarily responsible for paying the financial costs associated with climate change?** |  |  |  |  |  |  |
| Governments | 22 | 35 | 32 | 49 | 23 | 33 |
| Corporations | 39 | 35 | 16 | 22 | 17 | 32 |
| Individual citizens | 11 | 7 | 12 | 10 | 12 | 9 |
| All of the above/a combination | 28 | 21 | 14 | 16 | 16 | 21 |
| Other | 0 | 0 | 2 | 0 | 2 | 0 |
| None/Not happening | 0 | 0 | 0 | 0 | 27 | 2 |
| Not sure/refused | 0 | 1 | 24 | 3 | 4 | 2 |
| **Thinking now about companies that produce and distribute fossil fuels, do you support or oppose holding these companies accountable for a share of the financial costs of climate change?** |  |  |  |  |  |  |
| Strongly support | 64 | 46 | 31 | 10 | 10 | 41 |
| Somewhat support | 24 | 43 | 0 | 60 | 23 | 38 |
| Somewhat oppose | 5 | 6 | 15 | 23 | 11 | 9 |
| Strongly oppose | 5 | 4 | 7 | 5 | 48 | 8 |
| Not sure/refused | 2 | 1 | 47 | 2 | 9 | 4 |
|  |  |  |  |  |  |  |
| **Table S8. Demographics, by audience segment** | | | | | | |
|  | Alarmed | Concerned | Disengaged | Doubtful | Dismissive | Avg. |
| **Gender** |  |  |  |  |  |  |
| Male | 43 | 47 | 43 | 49 | 84 | 49 |
| Female | 57 | 53 | 57 | 51 | 16 | 51 |
| **What is your mother tongue?** |  |  |  |  |  |  |
| French | 25 | 27 | 19 | 24 | 10 | 24 |
| English | 65 | 59 | 60 | 65 | 76 | 63 |
| Other | 10 | 14 | 21 | 11 | 14 | 13 |
| **In which province do you currently live?** |  |  |  |  |  |  |
| Alberta | 8 | 8 | 6 | 19 | 24 | 11 |
| British Columbia | 13 | 14 | 13 | 10 | 16 | 13 |
| Prairies | 6 | 4 | 8 | 11 | 13 | 7 |
| Atlantic | 9 | 7 | 15 | 7 | 5 | 7 |
| Ontario | 39 | 41 | 30 | 33 | 30 | 38 |
| Quebec | 25 | 27 | 28 | 21 | 12 | 24 |
| **If a federal election were held today, for which party are you most likely to vote?** |  |  |  |  |  |  |
| Liberal Party of Canada | 36 | 36 | 21 | 23 | 8 | 31 |
| Conservative Party of Canada | 12 | 19 | 25 | 43 | 77 | 26 |
| The New Democratic Party | 12 | 13 | 0 | 8 | 0 | 10 |
| The Bloc Québécois | 4 | 3 | 2 | 1 | 0 | 3 |
| Green Party of Canada | 11 | 5 | 0 | 3 | 1 | 6 |
| Another party | 1 | 0 | 2 | 0 | 1 | 1 |
| I would not vote | 2 | 4 | 8 | 5 | 6 | 4 |
| Not sure/refused | 23 | 19 | 43 | 16 | 7 | 20 |
| **How interested are you in politics generally?** |  |  |  |  |  |  |
| Very interested | 50 | 24 | 8 | 21 | 57 | 32 |
| Somewhat interested | 36 | 51 | 36 | 44 | 34 | 44 |
| Not to interested | 9 | 17 | 30 | 21 | 7 | 15 |
| Not interested at all | 4 | 8 | 21 | 14 | 2 | 8 |
| Not sure/refused | 0 | 0 | 6 | 0 | 0 | 0 |
| **Do you usually consider yourself as being at the left, the right or the centre of the political spectrum?** |  |  |  |  |  |  |
| Right | 6 | 6 | 9 | 17 | 39 | 11 |
| Center right | 7 | 6 | 4 | 13 | 14 | 8 |
| Centre | 32 | 42 | 17 | 42 | 27 | 37 |
| Centre left | 16 | 13 | 2 | 4 | 4 | 11 |
| Left | 28 | 17 | 9 | 9 | 2 | 17 |
| Not sure/refused | 12 | 15 | 58 | 16 | 14 | 16 |
| **What is the highest level of education you have attained?** |  |  |  |  |  |  |
| Less than high school | 3 | 4 | 28 | 9 | 5 | 6 |
| High school graduate | 16 | 19 | 30 | 24 | 17 | 19 |
| Some college or tech school | 8 | 12 | 15 | 16 | 16 | 12 |
| College graduate | 32 | 34 | 19 | 32 | 31 | 32 |
| Graduate or professional degree | 42 | 32 | 8 | 19 | 31 | 31 |
